# Annotations

**Published:** 1889-12-07

**Authors:** 


### Annotations.

**Martin Tupper.**

Mr. Martin Tupper is dead, and it may be presumed that "superior persons" will now cease to laugh at him. The loudest bursts of laughter, and they were very loud a good many years ago, came from young newspaper writers, who, as is well known, are by far the cleverest, most learned, and most universal and unquestionable in their judgments on all manner of things, "of all mankind who can write and read," as Clarendon has it. Tupper's "Proverbial Philosophy," by which he is best known, is undoubtedly peculiar. The average critical Briton does not object to a proverb, nor even to a little philosophy. But he likes the proverb as short as may be, as crammed with obvious common-sense as possible, and to have no suspicion whatever of any kind of sentimentality about it. Your Englishman is religious, and he is sentimental, but only at times. On week days he is at business. The business may be nothing more than that of foxhunting or presiding at a magistrates' meeting. But it is business, and as such precludes the admixture of any kind of irrelevant matter, either of a sentimental or even of a religious kind. But women admired Mr. Tupper. They understood him. They saw that he meant well, and that his "Philosophy" covered a great deal of common everyday ground, and lighted it up with a mild halo of gentle and correct feeling. Inasmuch as it did that it was a distinct gain. With us life is much too "hard" all round. With the man of business it is terribly hard. The City of London—and we presume other centres of business are the same—is absolutely adamantine. To the City man at midday "Proverbial Philosophy" was ludicrous. What! Use fifty words where five would serve your purpose! Incredible: and impossible! But the lady of the City lord, when hard pressed by the thousand and one cares of a suburban household, takes up Tupper, and finds soothing and balm in him. If he spreads out his not very masculine thought over a large space, so much the better. It is like thin bread and butter. If the bread of thought be thin it is well that the butter of words should be thin too, otherwise the completed result is neither pleasant nor wholesome. We predict for "Proverbial Philosophy" at least a limited immortality, if such a contradiction in terms may pass muster. As for Mr. Tupper himself, he has lived a wholesome life; has been, and still is, appreciated most by those who knew him best; and has died at a ripe old age, truly mourned and regretted by the gentler sort both of men and women.

**Beware of Chichester.**

A bishop is the shepherd of his flock, and that not merely by virtue of his name, but by the very terms of his office. If he sees his sheep poisoning each other's morals he speaks out. Why should he not also speak out when he sees them poisoning each other's stomachs, and sending one another by the short cut of nauseous and deadly abominations to "that undiscovered country from whose bourne no traveller returns"? The Right Reverend, and right reasonable, Dr. Durnford, the venerable Bishop of Chichester, has for the second time during his long tenure of office lifted up his voice against the madness and public crime of his cathedral city. He makes an indictment against it, which probably some of its sage and self-satisfied town councillors think no more of than they would of a scolding husband's protest against Monday's soapsuds. But what strikes the pompous and empty-headed town councillor as a mere incident in his municipal career, strikes the outside world as the exposure of a state of things which would be discreditable to savages and cannibals, but which to the inhabitants of an ancient cathedral city is disgraceful and scandalous beyond the power of words. Those Englishmen who are not inhabitants of Chichester would hardly believe, except on the word of a bishop, that what Dr. Durnford stated at a public meeting of the principal inhabitants a short time ago was literally true. It seems that Chichester has within reach a water supply second to none in the kingdom. And yet, says the bishop, "the poorer classes are compelled to drink water that is absolutely unwholesome; very often teeming with visible animalculæ, and it is not necessary to use a microscope in order to see worms and unclean things floating on its surface. . . Chichester, which ought to have the best water in the county, is content with the very worst supply the county possesses. I believe," continued the virtuously indignant prelate, "that there are many wells in this city absolutely putrid, giving forth water unfit for the commonest domestic purposes, which stinks as it comes from the well." Chichester, it seems, is built, like St. Petersburg, to a certain extent on marshes; but whilst the marshes of St. Petersburg are more or less natural and more or less pure, the marshes of Chichester are entirely artificial, and consist of cesspools, or of cesspools and wells into which the cesspools empty themselves of their more fluid contents. This must be delightful for those who live in Chichester. There can be no necessity for eau de Cologne or lavender water there. The natural odours of the place must be so ever-present and so pungent as to make rivals impossible. The writer has faced the manifold "stinks" of various continental towns, but he hopes that no malign fate will compel him to visit Chichester. It is stated that the death-rate from zymotic diseases is habitually higher in this beautifully-situated cathedral city than in any one of the largest manufacturing towns in England. If that be so, the smallest tradesman in the city ought to be personally ashamed of the fact. For ourselves, we have no interest in the question except from a public point of view. So long as the cesspools and wells of the place remain what they are, it is clearly the duty of every journalist who takes thought for his fellow-countrymen to make his voice heard in every corner of the kingdom, bidding both great and small "Beware of Chichester."

**Bad Smells.**

An alarming occurrence is reported from the Bolton Woods Board School, Bradford, Yorkshire. Miss Williamson, a pupil teacher, shortly after commencing the work of her girls' class, was seen to fall suddenly forward on her face on the floor. When picked up she was found in a semi-conscious condition, and had to be taken home. Within a few minutes five of the children of the class also fell in the same way, and had to be removed from the school building. During the morning others were similarly affected, and by the time that the dinner hour had arrived no fewer than fourteen of the children had been taken home. They all complained of pains in the head, sudden sickness, and shivering fits. They all recovered in the course of the day. There seems to be some doubt on the spot as to the cause of the extraordinary occurrence. There need not be. If the event had happened in a private house, or in a public boarding-place, where all the inmates had partaken of the same kind of food, then there might have been some uncertainty. But in this case it is certain that most of the sufferers came from different houses, had partaken of different breakfasts, and were in every physiological respect under entirely different conditions. Therefore the cause must have been in the air which the fourteen were inhaling, and therefore, of course, the air must have been poisonous and bad. It is not stated that there was any offensive odour perceptible. Probably there was not, or an outcry would have been made about it. But, even though no perceptibly offensive smell was present, it is clear that there was a poison in the air, and that of a most powerful kind. Mark the striking and definite effect, so similar as to be identical in each case: sudden headache, cold shiverings, and such a degree of immediate prostration as to make fourteen healthy young persons fall prone upon the floor. Can anything more demonstrative be desired in the way of proof of the power of the poison? And yet these consequences were all caused by a bad smell, so mild in odour that it could not certainly be appreciated by the nose. Many persons think bad smells of little account, and consider that unless poisonous matter be taken as food or drink, no harm can be done. It is an absurdly unscientific view of the case, and both dangerous and fatal in its results. There are certain well-known poisons that act more powerfully when inhaled than in any other way. Some bad smells belong to this class. It is impossible to make the warning against all sorts of impure air too decisive or too urgent.

